# Burned-out testicular tumor with retroperitoneal lymph node metastasis: a case report

**DOI:** 10.4076/1752-1947-3-8705

**Published:** 2009-07-31

**Authors:** Stylianos Kontos, Grigorios Doumanis, Maria Karagianni, Vasilios Politis, Vasileios Simaioforidis, Stefanos Kachrilas, Sotirios Koritsiadis

**Affiliations:** 1 Department of Urology, General Hospital of Nikea, Mantouvalou St, Nikea, Piraeus 18454, Greece; 2 Department of Pathology, General Hospital of Nikea, Mantouvalou St, Nikea , Piraeus 18454, Greece

## Abstract

**Introduction:**

A burned-out seminoma of the testis is an exceptionally rare clinical entity, with few reports found in the literature.

**Case presentation:**

A case of burned-out tumor of the testis in a 31-year-old man is reported. The tumor presented as a retroperitoneal mass with histological characteristic of a seminoma. The testes on clinical examination were normal, and a suspicious lesion in the scrotum was only identified after ultrasound. Incision of the abdominal mass was decided, followed by orchectomy. Histological examination of the testis revealed a suspicious lesion with characteristics of spontaneous regression of germ cell tumors.

**Conclusion:**

We describe one of very few cases worldwide, where spontaneous regression of a primary testicular tumor occurred after demonstration of retroperitoneal lymph node metastasis, a phenomenon known as burned-out seminoma, which is hard to recognize and incompletely characterized by physicians.

## Introduction

Seminoma is the most frequent carcinoma of the testicle in the fourth decade of life and constitutes 60% to 65% of germ cell neoplasias. Several histopathologic characteristics of the tumor have been evaluated and three types of pure seminoma have been described as follows: a) classic, b) anaplastic and c) spermatocytic.

Internationally, three clinical stages for the determination of the extension of the tumor are admissible. Stage I is where the tumor is limited to the testis with or without invasion of epididymis or the spermatic cord. In Stage II the tumor has retroperitoneal lymph node metastases. Finally, in Stage III the tumor has distant metastases.

The germ cell tumor often gives lymph node metastasis, except from choriocarcinoma, which in an aggressive fashion is characterized by early hematogenous spread. We describe one of the very few cases worldwide where the spontaneous regression of a primary testicular tumor occurred after demonstration of retroperitoneal lymph node metastasis, a phenomenon known as burned-out seminoma.

## Case presentation

**Figure 1:**
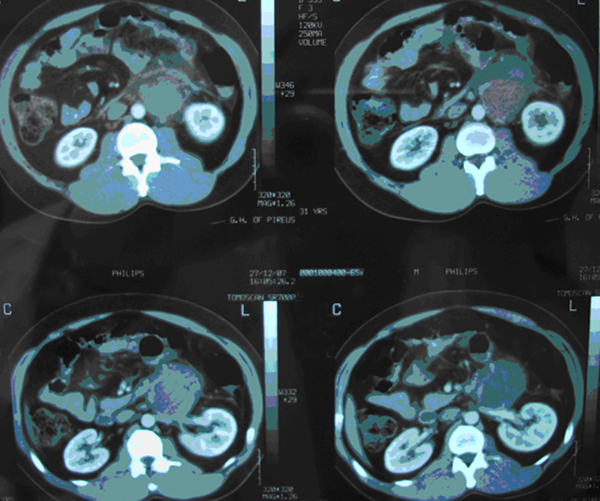
**Computed tomography scan showing a 7 cm retroperitoneal tumor on the left side of the aorta, compressing the left renal vein**.

A 31-year-old man presented with symptoms of incomplete ileus (diffuse abdominal discomfort, nausea, vomiting and abdominal distension). The patient had no relevant past medical history. Abdominal CT scan showed a retroperitoneal mass of 7 × 7 × 5 cm extending along the left side of the aorta, compressing but not completely occluding the left renal vein (Figure 1). There were also lymph nodes with varied diameter around the mass, and regions with necrosis and calcifications. Physical examination, especially of the testes, was normal. The patient's serum α-fetoprotein (α-FP), β human chorionic gonadotrophin (β-hCG) and lactate dehydrogenase (LDH) levels were normal, but scrotal ultrasonography (SUS) showed a normal right testis, while the left testis was small and contained an 8 mm suspicious hypo-echoic area associated with calcifications.

**Figure 2:**
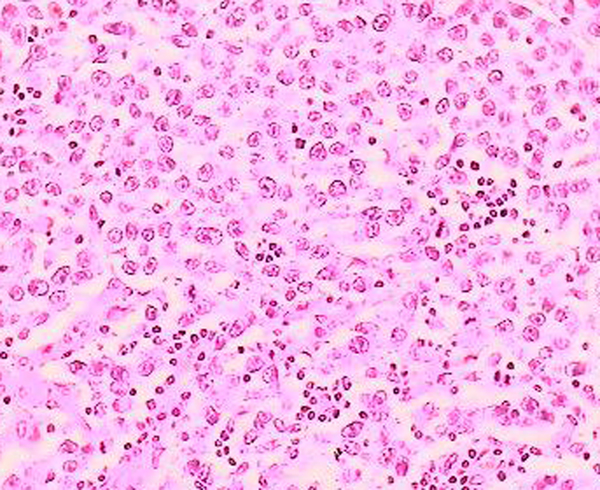
**Retroperitoneal mass with seminomatous germ cells (hematoxylene-eosine, ×40)**.

**Figure 3:**
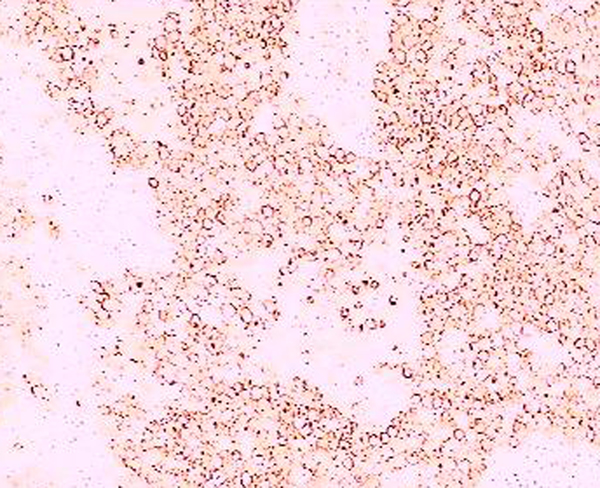
**Retroperitoneal mass with positive immunoreactivity to PLAP (placental alkaline phosphatase, ×40)**.

The patient had extensive resection of the retroperitoneal mass and lymphadenectomy. Histological examination showed a mass with a thin peripheral necrotic zone and associated dystrophic calcifications and persistent fibrous and inflammatory areas. The neoplastic cells had lymphoplasmacytic and eosinophilic infiltration, which varied in intensity, and numerous nuclear mitotic actions (Figure 2). Immunohistochemical analysis of the neoplastic cells showed a positive reaction to placental alkaline phosphatase (PLAP), showing a metastasis of a pure seminoma (Figure 3).

**Figure 4:**
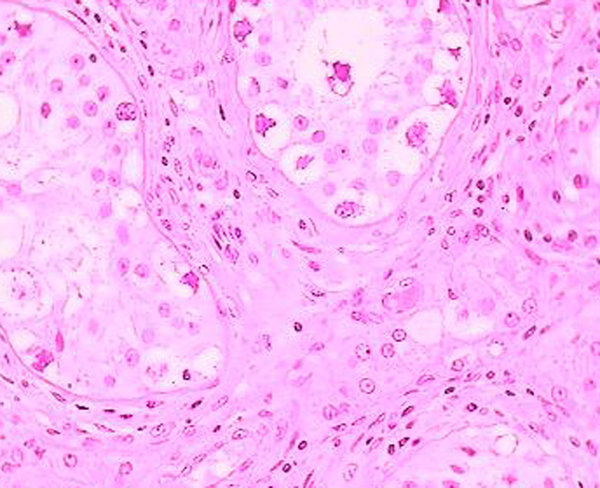
**Testicular scar, with seminomatous germ cell neoplasia (hematoxylene-eosine ×60)**.

**Figure 5:**
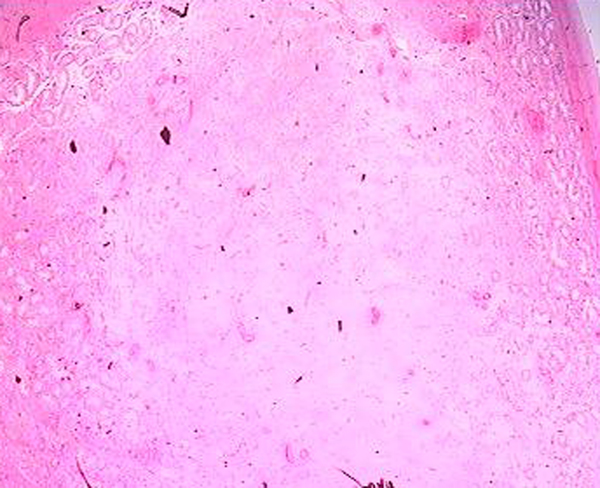
**Well-demarcated nodular scar with large aggregates of Leydig cells in the surrounding atrophic testis (hematoxylene - eosine ×40)**.

Histological examination of the testis after radical left orchidectomy showed a well-demarcated nodular scar. Many tubules had a Sertoli cell only pattern and others were completely hyalinized. Increased vascularity was manifested by a collection of small, atrophic, non-arborizing vessels. Testicular atrophy was demonstrated by shrunken tubules with decreased or absent spermatogenesis and thickened peritubular basement membranes peripheral to the scar (Figures 4 and 5). Testicular scarring and atrophy are diagnostic evidence of germ cell tumor regression in burned-out seminoma. The patient underwent additional chemotherapy.

## Discussion

The phenomenon of spontaneous regression of cancer has not been fully elucidated; it may possibly be defined as partial or complete disappearance of the tumor with no therapy and has been described in renal tumours, breast carcinoma, lymphoma and malignant melanoma [1].

Burned-out seminoma is the spontaneous regression of a testicular germ cell tumor with or without metastasis. In our case report, the primary testicular tumor with histological characteristics of a seminoma regressed after the development of metastasis in the retroperitoneum. Several immunological and ischemic mechanisms have been suggested to explain this spontaneous regression [2], but immunological mechanisms appear to be the most likely mechanism [3]. One hypothesis suggests that common tumor antigens can be recognized after repeated exposure, by cytotoxic T lymphocytes, which are subsequently replaced by fibrosis. This hypothesis of immunological mechanism [4] in the regression of the primary testicular tumor after metastasis, as well as its therapeutic consequences, is not proven but should be considered. Histological features that are helpful in establishing a diagnosis of a regressed testicular germ cell tumor include, apart from the scar formation, intratubular calcifications, lymphoplasmacytic infiltrate, hemosiderin-containing macrophages and testicular atrophy [5]. Standard cisplatinum-based chemotherapy, after removing the primary testicular tumor, is the basis of the treatment strategy [6]. The prognosis is excellent in cases of patients with an extragonadal germ cell tumor with histological patterns of seminoma in either the mediastinum or retroperitoneum, compared with cases of patients with a nonseminomatous extragonadal germ cell tumor. Unfortunately, the majority of patients with extragonadal germ cell tumors (80%) have a nonseminomatous extragonadal germ cell tumor and thereby have a poor prognosis [7].

## Conclusion

In patients who present with a retroperitoneal mass, diagnosis of metastatic progression of a germ cell neoplasia should be considered. A burned-out testicular tumor shows a distinctive constellation of findings that usually permits its recognition, which is significant because pure seminomatous germ cell tumors have a long term chance of cure, irrespective of the primary tumor site.

## Abbreviations

CT: computed tomography; α-FP: α-fetoprotein; β-hCG: β human chorionic gonadotrophin; LDH: lactate dehydrogenase; PLAP: placental alkaline phosphatase; SUS: scrotal ultrasonography.

## Consent

Written informed consent was obtained from the patient for publication of this case report and accompanying images. A copy of the written consent is available for review by the Editor-in Chief of this journal.

## Competing interest

The authors declare that they have no competing interests.

## Authors' contributions

All authors contributed to conceptualizing and writing this case report.
